# Gliotoxin Enhances Autophagic Cell Death via the DAPK1-TAp63 Signaling Pathway in Paclitaxel-Resistant Ovarian Cancer Cells

**DOI:** 10.3390/md17070412

**Published:** 2019-07-12

**Authors:** Ga-Bin Park, Jee-Yeong Jeong, Daejin Kim

**Affiliations:** 1 Department of Biochemistry, Kosin University College of Medicine, Busan 49267, Korea; 2 Department of Anatomy, Inje University College of Medicine, Busan 47392, Korea

**Keywords:** gliotoxin, DAPK1, TAp63, autophagy, drug resistance, ovarian cancer

## Abstract

Death-associated protein kinase 1 (DAPK1) expression induced by diverse death stimuli mediates apoptotic activity in various cancers, including ovarian cancer. In addition, mutual interaction between the tumor suppressor p53 and DAPK1 influences survival and death in several cancer cell lines. However, the exact role and connection of DAPK1 and p53 family proteins (p53, p63, and p73) in drug-resistant ovarian cancer cells have not been studied previously. In this study, we investigated whether DAPK1 induction by gliotoxin derived from marine fungus regulates the level of transcriptionally active p63 (TAp63) to promote apoptosis in an autophagy-dependent manner. Pre-exposure of paclitaxel-resistant ovarian cancer cells to gliotoxin inhibited the expression of multidrug resistant-associated proteins (MDR1 and MRP1-3), disrupted the mitochondrial membrane potential, and induced caspase-dependent apoptosis through autophagy induction after subsequent treatment with paclitaxel. Gene silencing of DAPK1 prevented TAp63-mediated downregulation of MDR1 and MRP1-3 and autophagic cell death after sequential treatment with gliotoxin and then paclitaxel. However, pretreatment with 3-methyladenine (3-MA), an autophagy inhibitor, had no effect on the levels of DAPK1 and TAp63 or on the inhibition of MDR1 and MRP1-3. These results suggest that DAPK1-mediated TAp63 upregulation is one of the critical pathways that induce apoptosis in chemoresistant cancer cells.

## 1. Introduction

Death-associated protein kinase-1 (DAPK1) is a Ca^2+^/calmodulin (CaM)-regulated serine/threonine kinase that mediates cell death [1]. Downregulation of DAPK1 by methylation in its promoter region is detected in various cancers, including pancreas, lung, and head and neck. Low DAPK1 expression in these tumors is closely related to frequent lymph node metastasis and poor clinical outcomes [2,3,4]. Overexpression or activation of DAPK1, as a tumor suppressor, is involved in cell death. Ectopic DAPK expression induces p53-dependent apoptosis in mouse embryo fibroblasts [5], and tumor necrosis factor-alpha (TNF-α) and interferon-gamma (IFN-γ) induce DAPK1 expression through inhibiting NF-κB activity [6]. DAPK1 activation not only requires cell cycle arrest and caspase-dependent apoptosis [7] but also contributes to autophagic cell death by reducing the interaction between Beclin-1 and Bcl-2 and Bcl-X_L_ [8]. Furthermore, DAPK-1 induces caspase-independent cell death through activating autophagosome formation [9]. However, DAPK1 sometimes antagonizes apoptosis in a cell-type-dependent manner. DAPK1 depletion using antisense DAPK1 cDNA promotes caspase-mediated apoptosis via TNF [10], and DAPK1 plays an essential role in the proliferation of p53-mutant estrogen receptor-negative breast cancer cells [11]. These results demonstrate that the role and molecular mechanism of DAPK1 in human cancers are not clearly understood. 

Tumor suppressor p53 expression responds to various types of cellular damage and stress, including oncogenic stimuli [12,13]. Cells expressing wild-type p53 experience DAPK1 upregulation after stimulation with anticancer drugs or UV exposure [14]. In contrast, DAPK1 overexpression promotes p53 expression, resulting in the suppression of oncogenic transformation [5]. p53 mutations that reduce or abolish its function are closely associated with anticancer drug resistance in various cancers [15,16], whereas mutant p53 occurrence in ovarian cancer has no effect on apoptotic death induced by paclitaxel [17]. Ovarian cancer co-expressing p53 and Bcl-2 has been shown to have the best response to paclitaxel chemotherapy [18]. These contradictory results suggest that p53 and DAPK1 influence several different molecular pathways to induce cancer cell death, and the mutual relationship between DAPK1 and p53 is dependent on cell type and cell conditions.

The p53 family is composed of three homologous proteins, p53, p63, and p73. These proteins share essential structural domains and have similar cellular functions in proliferation, differentiation, tumorigenesis, and death [19,20]. Transcriptionally active p63 (TAp63), a p53 isoform, plays a critical role in not only intracellular fatty acid generation [21] but also tumor suppression [22] and metastasis prevention [23]. We have previously reported that TAp63 activation induces apoptosis in Epstein-Barr virus (EBV)-transformed B cells after treatment with baicalein [24], and TAp63 expression in TLR4-stimulated colon cancer cells promotes fatty acid-mediated metastasis [25]. However, the precise relationship between DAPK1 and TAp63 in modulating apoptosis in cancer is unclear, and the autophagy-related signaling pathway regulated by TAp63 in cancer needs to be investigated. 

Gliotoxin, a secondary metabolite of marine fungus *Aspergillus fumigatus*, is not only a member of the epipolythiodioxopiperazine family but also characterized by a disulfide bond across a piperazine ring and an aromatic amino acid [26,27]. Treatment with gliotoxin of mouse immune cells inhibits nuclear factor kappa B (NF-κB), resulting in the downregulation of inflammatory genes activation [28]. Reactive oxygen species (ROS) induced by gliotoxin contribute to suppression of NF-κB, leading to the apoptosis of human fibrosarcoma cell line (HT1080) [29]. Gliotoxin induces apoptosis in various human cancers cells, including uterine cervix cancer cell line (Hela), chondrosarcoma cell line (SW1353), chronic lymphocytic leukemia cell, and breast cancer cell line (MCF-7) [30,31,32]. Recently, it has been reported that gliotoxin treatment induces apoptotic death in doxorubicin-resistant lung cancer cells through disrupting mitochondrial function and activating p53 downstream target molecules [33]. However, the expression and specific role of DAPK1 in ovarian and paclitaxel-resistant ovarian cancer cells after treatment with gliotoxin have not been studied. In this study, we investigated whether DAPK1 regulates apoptotic death in paclitaxel-resistant ovarian cancer cells and examined the relationship between DAPK1 and p53 family proteins in inducing autophagic cell death after treatment with gliotoxin.

## 2. Results

### 2.1. Treatment with Gliotoxin Suppresses Growth and Reduces Resistance in Paclitaxel-Resistant Ovarian Cancer Cells

**Figure 1:**
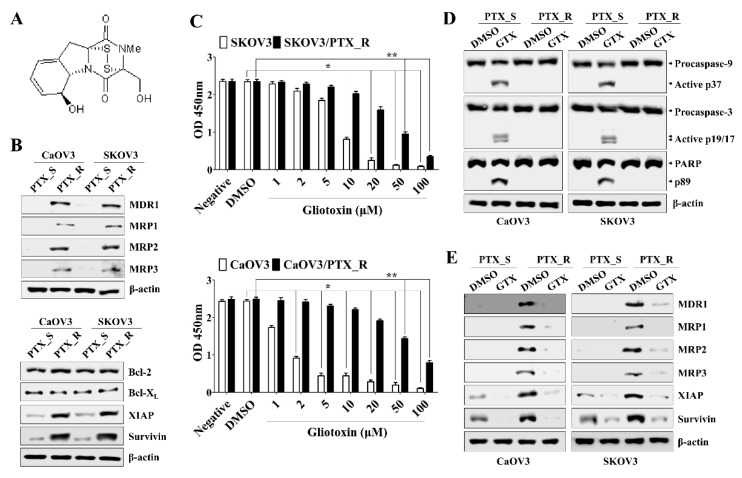
Treatment with gliotoxin (GTX) suppressed cell growth and reduced resistance in paclitaxel-resistant ovarian cancer cells. (**A**) The chemical structure of gliotoxin used in the whole experiment in this study. (**B**) Total protein from each group of PTX-sensitive cells (PTX_S) and PTX-resistant cells (PTX_R) was analyzed by Western blotting with the indicated antibodies. The expressions of multidrug resistant-associated proteins (MDR1-3), X-linked inhibitor of apoptosis protein (XIAP), and surviving were increased in CaOV3/PTX_R and SKOV3/PTX_R cells (**C**) Cells were treated with the indicated drug concentration for 24 h. Cell viability was measured using a Cell Counting Kit-8 assay. The absorbance at 450 nm is presented. n = 3. *p < 0.001 (GTX-treated PTX_S ovarian cancer cells vs. DMSO-treated PTX_S ovarian cancer cells); **p < 0.001 (GTX-treated PTX_R ovarian cancer cells vs. DMSO-treated PTX_R ovarian cancer cells). (**D**,**E**) Cells (1.5 × 10^5^/well) were treated with 5 μM GTX for 24 h. Total protein was subjected to Western blot analysis with the indicated antibodies. β-actin served as an internal control. Treatment with GTX of PTX_R ovarian cancer cells reduced the expression of MDR1-3, XIAP, and surviving, but not the cleavage of caspase-9 (active p37/35) and caspase-3 (active p19/17). The results are representative of three independent experiments.

We first determined the 50% inhibitory concentration (IC*50*) value of paclitaxel in ovarian cancer cells using Cell Counting Kit-8 (CCK-8) assays as described in the Methods section. The IC50 values of paclitaxel in CaOV3 and SKOV3 cells were 2.71 ± 0.01 nM and 5.15 ± 0.02 nM, respectively, at 48 h. Established paclitaxel-resistant ovarian cancer cells showed profound morphological differences compared to parental cell lines under microscope observation (Supplemental Figure S1A). Additionally, paclitaxel-resistant CaOV3 cells (CaOV3/PTX_R) and SKOV3 cells (SKOV3/PTX_R) sustained their proliferation rates after exposure to a high dose (100 nM) of paclitaxel (Supplemental Figure S1B). We next investigated whether treating chemoresistant ovarian cancer cells with gliotoxin prevents cell growth and induces apoptosis. Chemoresistant ovarian cancer cells not only upregulated multidrug resistant-associated proteins (MDR1 and MRP1-3) but also induced the expression of anti-apoptotic proteins, including X-linked inhibitor of apoptosis protein (XIAP), and cell survival (Figure 1A). Although exposure to gliotoxin slightly prevented the proliferation of CaOV3/PTX_R and SKOV3/PTX_R cells (Figure 1B), treating drug-resistant ovarian cancer cells with gliotoxin failed to induce cleaved caspase-9 (active p37) and caspase-3 (active p19/17) or the downstream target cleaved poly (ADP-ribose) polymerase (PARP) (Figure 1C). However, the levels of drug-resistant proteins were markedly decreased after treatment with gliotoxin (Figure 1D). These results suggest that gliotoxin renders the chemoresistant ovarian cancer cells vulnerable to cytotoxic agents, even though the drug alone could not induce the apoptotic death of cancer cells.

### 2.2. Sequential Treatment with Gliotoxin Followed by Paclitaxel Promotes Apoptotic Death in Paclitaxel-Resistant Ovarian Cancer Cells

**Figure 2:**
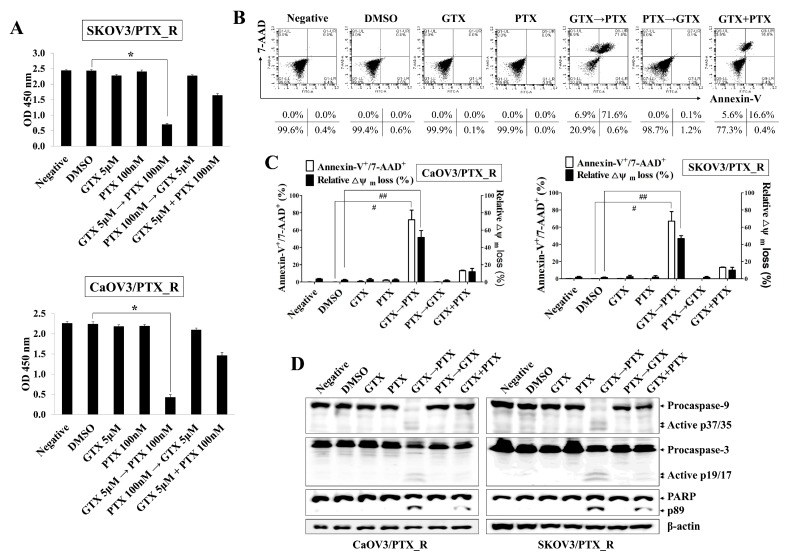
Sequential treatment with gliotoxin followed by paclitaxel induces apoptotic death in paclitaxel-resistant ovarian cancer cells. Cells were seeded into 96-well plates (1 × 10*^4^* cells/well) or 6-well plates (1.5 × 10*^5^* cells/well) and pre-treated with GTX (5 μM) for 8 h followed by PTX (100 nM) for 48 h. For comparison, untreated control cells were cultured with media in the presence of DMSO. (**A**) Cell viability was measured using a Cell Counting Kit-8 assay. The absorbance at 450 nm is presented. n = 3. *p < 0.001 (PTX_R ovarian cancer cells treated with GTX followed by PTX vs. DMSO-treated PTX_R ovarian cancer cells). (**B**,**C**) To determine the degree of apoptosis, cells were stained with annexin-V-FITC and 7-AAD and analyzed by flow cytometry. Dot-plot graphs show the percentage of viable cells (annexin-V*^-^*/7-AAD*^-^*), early-stage apoptotic cells (annexin-V*^+^*/7-AAD*^-^*), late-stage apoptotic cells (annexin-V*^+^*/7-AAD*^+^*), and necrotic cells (annexin-V*^-^*/7-AAD*^+^*). Late-stage apoptotic cells (annexin-V+/7-AAD+) were evaluated by flow cytometry. # *p* < 0.005 (PTX_R ovarian cancer cells treated with GTX, followed by PTX vs. DMSO-treated PTX_R ovarian cancer cells). To measure Δψ*_m_* disruption, cells were stained with DiOC*_6_*. Diminished DiOC*_6_* fluorescence (%) indicates Δψ*_m_* disruption. ## *p* < 0.005 (PTX_R ovarian cancer cells treated with GTX, followed by PTX vs. DMSO-treated PTX_R ovarian cancer cells). Each value is expressed as the mean ± SD from three independent experiments (n = 3). (**D**) Whole cell lysates were subjected to Western blot analysis using the indicated antibodies. Sequential treatment with GTX followed by PTX induced the activated caspase-9 (active p37/35) and caspase-3 (active p19/17) in PTX_R ovarian cancer cells. β-actin served as an internal control. The results are representative of three independent experiments.

As shown in Figure 1B, treatment with 5 μM GTX not only started to prevent the proliferation of PTX-sensitive SKOV3 cells but also blocked the growth of CaOV3/PTX_R and SKOV3/PTX_R cells. Furthermore, exposure to 5 μM GTX reduced in MDR1 and MRP1-3 expression in CaOV3/PTX_R and SKOV3/PTX_R cells, but not the induction of active form caspase-9 and caspase-3. We also observed that the exposure to 100 nM paclitaxel for 48 h induced nearly completely blocked the proliferation of PTX-sensitive ovarian cancer cells, whereas the growth rate of CaOV3/PTX_R and SKOV3/PTX_R cells was preserved (Figure S1). Based on these results, we next investigated whether co-treatment with gliotoxin and paclitaxel promotes apoptotic death in drug-resistant ovarian cancer cells. To verify the sensitizing effect of gliotoxin to the anti-cancer drug through reducing MDR1 and MRP1-3 in paclitaxel-resistant ovarian cancer cells, CaOV3/PTX_R and SKOV3/PTX_R cells were pre-exposed to gliotoxin (5 μM) for 8 h and then sequentially treated with paclitaxel (100 nM) for 48 h. Consecutive treatment with gliotoxin and paclitaxel significantly prevented CaOV3/PTX_R and SKOV3/PTX_R cell growth compared to co-treatment and reverse sequential treatment (Figure 2A). When CaOV3/PTX_R and SKOV3/PTX_R cells were treated with gliotoxin, and then paclitaxel, the apoptotic death of chemoresistant ovarian cancer cells was synergistically increased (Figure 2B,C). Furthermore, drug-resistant ovarian cancer cells treated with gliotoxin followed by paclitaxel exhibited activation and cleavage of caspase-9, caspase-3, and PARP (Figure 2D). These results suggest that pre-exposure to gliotoxin reverses paclitaxel resistance in chemoresistant ovarian cancer cells via the induction of apoptotic death by chemotherapeutic agents.

### 2.3. Pre-Exposure to Gliotoxin Followed by Paclitaxel Upregulates the TAp63 Expression, Leading to the Caspase-Dependent Apoptosis in Drug-Resistant Ovarian Cancer Cells

**Figure 3:**
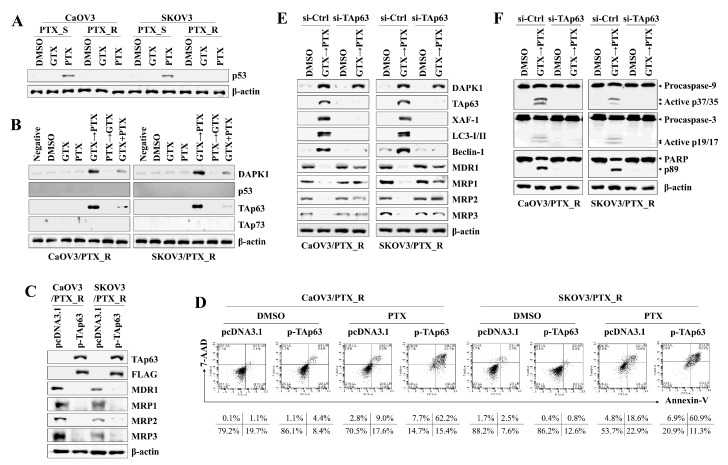
Pre-exposure to gliotoxin followed by paclitaxel induces caspase-dependent apoptosis in drug-resistant ovarian cancer cells by upregulating TAp63 expression. (**A**,**B**) Cells were seeded into 6-well plates (1.5 × 10*^5^* cells/well), pre-treated with GTX (5 μM) for 8 h and then treated with PTX (100 nM) for an additional 48 h. For comparison, untreated control cells were cultured with media in the presence of DMSO. Total protein was subjected to Western blot analysis with the indicated antibodies. The cells treated with GTX followed by PTX upregulated the expression of DAPK1 and TAp63. β-actin served as an internal control. (**C**,**D**) Cells were transfected with either empty vector pcDNA3.1 or TAp63 expression vector. (**C**) Transfection efficiency was determined by immunoblot using TAp63 and FLAG antibodies. Whole cell lysates were analyzed by Western blotting using the indicated antibodies. Overexpression of TAp63 downregulated the levels of MDR1 and MRP1-3. (**D**) Percentages of apoptotic cells were analyzed by annexin-V/7-AAD staining. The number of late-stage apoptotic cells (annexin-V*^+^*/7-AAD*^+^*) was calculated by flow cytometry. (**E**,**F**) Cells (1.5 × 10*^5^*/well) were pre-treated with GTX (5 μM) for 8 h and then treated with PTX (100 nM) for an additional 24 h. Then, the cells were transfected with 200 nM siRNA against TAp63 or control. Cells were used for further experiments 40 h after transfection. The cells were analyzed by Western blotting with the indicated antibodies. Targeted inhibition of TAp63 suppressed the expression of autophagosome-related LC3-I/II and Beclin-1 (E) and prevented the upregulation of activated caspase-9 (active p37/35) and caspase-3 (active p19/17) for apoptotic death (**F**). β-actin served as an internal control. The results are representative of three independent experiments.

We next investigated the underlying signaling pathway to determine the role and relationship of DAPK1 and p53 family proteins. Although p53 expression was upregulated in CaOV3 and SKOV3 cells after paclitaxel stimulation (Figure 3A), exposure to gliotoxin or paclitaxel failed to induce p53 expression in CaOV3/PTX_R and SKOV3/PTX_R cells (Figure 3B). In contrast, cells treated with gliotoxin followed by paclitaxel had increased expression of DAPK1 and TAp63, a p53 family member (Figure 3B). To determine the role of TAp63 in the apoptotic death of CaOV3/PTX_R and SKOV3/PTX_R cells, we transfected TAp63-expressing plasmids into drug-resistant ovarian cancer cells. The expression of drug resistance-associated proteins was prominently lower in CaOV3/PTX_R and SKOV3/PTX_R cells with forced TAp63 expression than in cells transfected with empty vector (Figure 3C). Furthermore, TAp63 overexpression led to apoptosis in paclitaxel-resistant ovarian cancer cells after treatment with paclitaxel (Figure 3D). Sequential treatment with gliotoxin followed by paclitaxel induced the expression of autophagosome-related molecules (LC3-I/II and Beclin-1) and the apoptosis-related protein, Bax (Figure S2). Although TAp63 gene silencing had no effect on DAPK1 expression, reduced TAp63 expression prevented the induction of XIAP-associated factor 1 (XAF1), LC3-I/II, and Beclin-1, as well as the reductions in MDR1 and MRP1-3 expression, after sequential treatment with gliotoxin and paclitaxel (Figure 3E). Targeted inhibition of TAp63 also prevented the expression of cleaved and activated caspase-9, caspase-3, and PARP in CaOV3/PTX_R and SKOV3/PTX_R cells after treatment with gliotoxin followed by paclitaxel (Figure 3F). These results suggest that TAp63 expression plays an important role in enhancing apoptosis through the downregulation of multidrug resistant-associated proteins.

### 2.4. DAPK1/TAp63-Mediated Autophagy Regulates Apoptotic Death in Drug-Resistant Ovarian Cancer Cells after Consecutive Treatment with Gliotoxin and Paclitaxel

**Figure 4:**
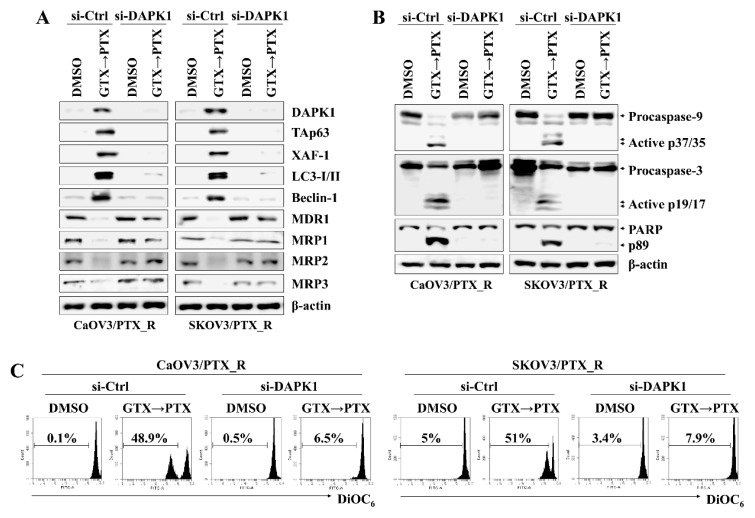
Increased DAPK1 induced by pre-exposure to gliotoxin and paclitaxel upregulates TAp63 expression and autophagy signaling in drug-resistant ovarian cancer cells. The cells (1.5 × 10*^5^*/well) were pre-treated with GTX (5 μM) for 8 h and then treated with PTX (100 nM) for an additional 24 h. Next, cells were transfected with 200 nM siRNA against TAp63 or control. Cells were used for further experiments 40 h after transfection. (**A**,**B**) The cells were analyzed by Western blotting with the indicated antibodies. DAPK1 silencing prevented the activation of downstream target molecules, including transcriptionally active p63 (TAp63), XIAP-associated factor 1 (XAF-1), LC3-I/II, and Beclin-1 as well as the cleavage of caspase-9 (active p37/35) and caspase-3 (active p19/17) by treatment with gliotoxin followed by paclitaxel. β-actin served as an internal control. (**C**) To measure Δψ*_m_* disruption, cells were stained with DiOC6 and analyzed by flow cytometry. Diminished DiOC*_6_* fluorescence (%) indicates Δψ*_m_* disruption. The results are representative of three independent experiments.

**Figure 5:**
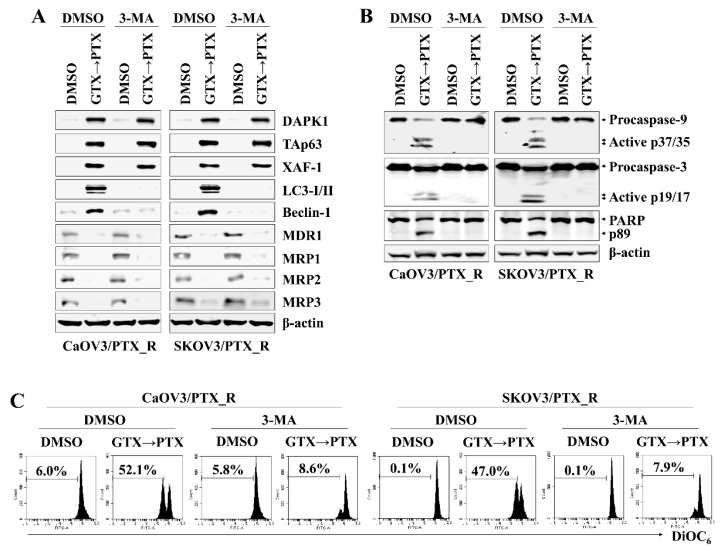
DAPK1/TAp63-mediated autophagy induction mediates apoptotic death in drug-resistant ovarian cancer cells after continuous treatment with gliotoxin and paclitaxel. (**A**–**C**) To inhibit autophagic signaling, cells (1.5 × 10*^5^*/well) were pre-exposed to 3-methyladenine (3-MA) (10 mM) for 2 h. Cells were pre-treated with GTX (5 μM) for 8 h and then treated with PTX (100 nM) for an additional 48 h. For comparison, untreated control cells were cultured with media in the presence of DMSO. (**A**,**B**) Whole cell lysates were subjected to Western blot analysis using the indicated antibodies. Pretreatment with 3-MA effective prevented the expression of autophagosome-related proteins (LC3-I/II and Beclin-1) and cleaved form of caspase-9 (active p37/35) and caspase-3 (active p19/17), but had no effect on downregulation of MDR-1 and MRP1-3 after treatment with gliotoxin followed by paclitaxel. β-actin served as an internal control. (**C**) To measure Δψ*_m_* disruption, cells were stained with DiOC*_6_* and analyzed by flow cytometry. Diminished DiOC*_6_* fluorescence (%) indicates Δψ*_m_* disruption. The results are representative of three independent experiments.

DAPK1 and p53 mutually induce apoptosis in cancer cells (6, 11). To determine the association between DAPK1 and TAp63 in inducing apoptosis in CaOV3/PTX_R and SKOV3/PTX_R cells after sequential treatment with gliotoxin and paclitaxel, we investigated the effect of DAPK1 on TAp63 expression, autophagosome-related molecule levels, and mitochondrial membrane potential changes. DAPK1-knockdown CaOV3/PTX_R and SKOV3/PTX_R cells exhibited suppressed TAp63, XAF-1, LC3-I/II, and Beclin-1 expression and downregulated MDR1 and MRP1-3 expression after sequential treatment with gliotoxin and then paclitaxel (Figure 4A). In addition, DAPK1 gene silencing using siRNA prevented the cleavage of caspase-9, caspase-3, and PARP (Figure 4B) as well as the depolarization of mitochondria membranes induced by treating paclitaxel-resistant ovarian cancer cells with gliotoxin followed by paclitaxel (Figure 4C). We finally investigated the connection between the DAPK1-TAp63 signaling pathway and autophagy-related cell death using 3-methyladenine (3-MA), an autophagy inhibitor. Pre-exposure of CaOV3/PTX_R and SKOV3/PTX_R cells to 3-MA had no effect on the expression of DAPK1, TAp63, and XAF1 after subsequent treatment with gliotoxin and paclitaxel (Figure 5A), whereas multidrug resistant-associated protein levels remained low (Figure 5A). Furthermore, 3-MA efficiently blocked the activation of the caspase-dependent apoptotic pathway and the depolarization of mitochondria membranes after sequential treatment with gliotoxin followed by paclitaxel (Figure 5B,C). These results suggest that DAPK1/TAp63-mediated autophagy is one of the key downstream target pathways that induce apoptosis in drug-resistant ovarian cancer cells and demonstrate that multidrug resistant-associated protein levels are regulated in an autophagy-independent manner after sequential treatment with gliotoxin followed by paclitaxel.

## 3. Discussion

**Figure 6:**
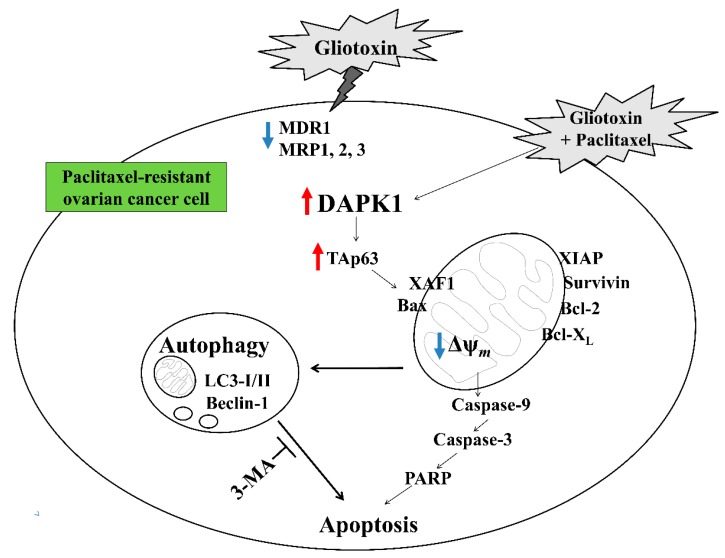
Schematic diagram of the intracellular signaling mechanism after sequential treatment with gliotoxin followed by paclitaxel in human ovarian cancer cells.

The tumor suppressor p53 promotes autophagy by inducing various autophagy-related genes, including DAPK1, a kinase acting in the early steps of autophagy [14,34]. DAPK1 overexpression also promotes the activation of cell death-associated signaling pathways, including autophagy-related apoptosis [1]. However, DAPK1 expression is frequently downregulated in B cell lymphoma and non-small cell lung cancer through multiple mechanisms, including promoter methylation [35,36]. TAp63, a p53 family member sharing a transactivation domain, has been reported to regulate the same target genes [19,20]. TAp63 not only inhibits cell growth but also prevents cell cycle progression in p53-deficient cancer cells [37]. These reports demonstrate that DAPK1 plays an important role in apoptosis induced by cytotoxic drug treatment and that the TAp63 and/or DAPK-related signaling pathways are promising candidates for controlling cancer growth in certain tumor environments. In this study, treatment with gliotoxin reversed the paclitaxel resistance of drug-resistant ovarian cancer cells through the downregulation of multidrug resistant-associated proteins. In addition, sequential treatment with gliotoxin followed by paclitaxel activated the DAPK1-mediated TAp63 signaling pathway to induce autophagic cell death in paclitaxel-resistant ovarian cancer cells (Figure 6). These results suggest that monitoring TAp63 and DAPK1 expression level is critical for detecting paclitaxel resistance and deciding whether to use paclitaxel in advanced or recurrent ovarian cancer patients. 

DAPK1 activation by cell death-inducing stimuli promotes apoptosis through the activation of p53-dependent p14/p19ARF tumor suppressor genes [5]. DAPK1 overexpression activated autophagic apoptotic death in a caspase-independent manner in breast and cervical cancer cells expressing wild-type p53 [9]. In contrast, stimulation with TGF-beta resulted in DAPK1-induced mitochondrial damage, leading to caspase-dependent apoptosis in a p53-depleted hepatoma cell line [38]. These contradictory results demonstrate that the role of DAPK1 might require further study to understand the connection with p53 or p53 family proteins in the apoptosis pathway. Although treating CaOV3 and SKOV3 cells with paclitaxel increased p53 expression, sequential exposure to gliotoxin followed by paclitaxel upregulated the levels of DAPK1 and TAp63 but not p53 and TAp73 expression in CaOV3/PTX_R and SKOV3/PTX_R cells. Furthermore, gene silencing of DAPK1 using siRNA in CaOV3/PTX_R and SKOV3/PTX_R cells prevented autophagy induction, caspase activation, and mitochondrial membrane disruption, as well as TAp63 activation. These results suggest that DAPK1 contributes to TAp63 activation to induce autophagic cell death in paclitaxel-resistant ovarian cancer cells after consecutive treatment with gliotoxin and paclitaxel. However, the precise association based on the molecular mechanism of DAPK1 and TAp63 in various cancer environments still needs to be investigated. 

Cells that survive previous chemotherapy obtain resistance to several anticancer drugs through their development of various defense mechanisms, including promoting drug efflux capability, altering drug metabolism, and changing drug targets [39]. Inactivation of p53 or mutant p53 in cancer cells decreases drug accumulation through the upregulation of multidrug-resistance protein (MRP1), which mediates ATP-dependent drug efflux [40]. Although exposure to gliotoxin induces mitochondrial membrane disruption and p53-dependent apoptotic cell death in adriamycin-resistant non-small cell lung cancer cells [33], the acquired mechanism to overcome the cytotoxic effects of chemotherapeutic drugs might be very diverse in cell type- or tumor environmental-dependent manners. In addition, the contribution of other p53 family proteins in the absence of wild-type p53 to overcome anticancer drugs is still unclear. Sequential treatment with gliotoxin, followed by paclitaxel increased the level of TAp63 in paclitaxel-resistant ovarian cancer cells. Furthermore, forced expression of TAp63 by transfection with a TAp63-containing plasmid reduced the expression of multidrug resistant-associated proteins (MDR1 and MRP1-3). These results suggest that TAp63 also plays an important role in modulating drug resistance without wild-type p53. 

Although pre-exposure to 3-MA before sequential treatment efficiently blocked cleaved caspase-9, caspase-3, and PARP generation and prevented mitochondrial membrane disruption, pretreatment with 3-MA still inhibited multidrug resistant-associated protein levels but failed to attenuate DAPK1-TAp63 signaling pathway activation. These results suggest that the DAPK1-TAp63 pathway controls autophagy induction and drug-resistant protein expression in an independent manner to promote the apoptotic pathway after sequential treatment with gliotoxin, followed by paclitaxel (Figure 6). 

Taken together, our results suggest that gliotoxin might be a promising agent to control advanced or recurrent ovarian cancer in clinical situations by reducing paclitaxel resistance. Our data also demonstrate that DAPK1 and TAp63 levels could be used as diagnostic or determining factors of drug resistance before starting repeated chemotherapy against ovarian cancer.

## 4. Materials and Methods 

### 4.1. Cell Lines and Reagents

Human ovarian cancer cell lines CaOV3 and SKOV3 (American Type Culture Collection (ATCC), Manassas, VA, USA) were used in this study. These cells were cultured in DMEM and McCoy’s 5A (Corning Incorporated, Corning, NY, USA) with 10% FBS (RMBIO, Missoula, MT, USA), glutamine, and antibiotics and maintained at 37 °C under 5% CO2. Gliotoxin (GTX) and 3-methyladenine (3-MA) were obtained from TOCRIS (Bristol, UK). Paclitaxel (PTX) was purchased from Sigma-Aldrich (St. Louis, MO, USA). Paclitaxel-resistant sublines (CaOV3/PTX_R and SKOV3/PTX_R) were established in the paclitaxel-sensitive (PTX_S) parent cell lines CaOV3 and SKOV3, respectively, by sequential exposure of cells to increasing concentrations of PTX 2.5 ~ 100 nM over 6 months. Finally, the authenticity of the drug-resistant sublines was confirmed by the ATCC Standards Development Organization (SDO) through short tandem repeat profiling in accordance with the American National Standards Institute (ANSI) Standard (ASN-0002). 

### 4.2. Proliferation Assay with Cell Counting Kit-8

Cell proliferation was measured using a Cell Counting Kit-8 (CCK-8) (Enzo Life Sciences, Farmingdale, NY, USA) as described in the supplier’s protocol. Cells were seeded into 96-well plates (1 × 10**^4^** cells/well) and pre-treated with GTX (5 μM) for 8 h and then treated with PTX (100 nM) for an additional 48 h. For comparison, non-treated control cells were cultured with media in the presence of DMSO. After drug treatment, the cells were stained with 10 μL of CCK-8 dye in 90 μL of culture medium for 2 h at 37 °C. The absorbance was measured at 450 nm.

### 4.3. Analysis of Apoptosis by Flow Cytometry

The percentages of cells undergoing apoptosis were measured by flow cytometry with fluorescein isothiocyanate (FITC)-labeled annexin-V (BD Biosciences, San Diego, CA, USA) and 7-amino actinomycin D (7-AAD) (BD Biosciences). The cells were suspended in 100 μL of 1× annexin-V binding buffer; then, FITC-conjugated annexin-V (3 μL) and 7-AAD (3 μL) were added to the suspensions, and the cells were kept at room temperature for 15 min in the dark. The stained cells were monitored with a BD Accuri**^TM^** C6 (BD Biosciences).

### 4.4. Measurement of Mitochondria Membrane Potential (△ψ_m_)

Changes in mitochondrial membrane potential were determined using DiOC**_6_** (3,3’-dihexyloxacarbocyanine iodide; Molecular Probes, Eugene, OR). Cells were seeded into 96-well plates (1 × 10**^4^** cells/well), pre-treated with GTX (5 μM) for 8 h, and then treated with PTX (100 nM) for an additional 48 h. Cells were harvested, washed twice with PBS, resuspended in PBS supplemented with DiOC**_6_** (20 nM), incubated in the dark at 37 °C for 15 min, and analyzed immediately using a flow cytometer with an FL-1 filter.

### 4.5. Western Blot Analysis

Harvested cells were lysed with RIPA buffer (Elpis Biotech, Daejeon, Korea) supplemented with a protease inhibitor cocktail and phosphatase inhibitors (Sigma-Aldrich). Equal amounts of protein (10 μg/sample) determined with a BCA assay kit (Pierce, Rockford, IL, USA) were subsequently loaded onto SDS-PAGE gels. After electrophoresis, the proteins were transferred onto nitrocellulose membranes (Millipore Corp., Billerica, MA, USA). The membranes were blocked with 5% non-fat skim milk and probed with primary antibodies. The expression level of target proteins was determined using a chemiluminescence kit (Advansta Corp., Menlo Park, CA, USA) and an Amersham Imager 600 (GE Healthcare Life Sciences, Little Chalfont, UK). The expression levels of β-actin were used as a control.

### 4.6. Small Interfering RNA (siRNA) Transfection

Human TAp63-siRNA (5’-GCA CAC AGA CAA AUG AAU UUU-3’), human DAPK1-siRNA (5’-CAA CTA TGA TGT TAA CCA A-3’), and negative control-siRNA (Cat. No. SN-1001-CFG) were obtained from Bioneer (Daejeon, Korea). Cells were seeded at a density of 1.5 × 10^5^ per well in a 6-well plate and grown overnight. The cells were then transfected with 200 nM siRNA using Lipofectamine RNAiMAX Reagent (Invitrogen, Carlsbad, CA, USA) as described in the supplier’s protocol. The cells were used for further experiments at 48 h after transfection.

### 4.7. TAp63 Overexpression Using Transient Transfection

Transient transfection of cultured cells was performed using Lipofectamine 2000 as described in the supplier’s instructions. Cells were plated on 6-well culture plates at a density of 2 × 10^5^ cells/well and transfected the next day. Typically, 10 ng of construct DNA was transfected with 9 μL of Lipofectamine. The cultured cells were transiently transfected with either a TAp63 expression vector of TAp63 cDNA cloned into pcDNA3.1 (Addgene, Cambridge, MA, USA) or empty vector pcDNA3.1 (Invitrogen). Cells were transfected for 48 h and analyzed by Western blotting.

### 4.8. Statistical Analysis

Student’s *t*-test and one-way analysis of variance (ANOVA) using SPSS version 24.0 statistical software (IBM Corp., Armonk, NY, USA) were used for all statistical analyses. The data are presented as the mean ± standard deviation (SD). Differences were determined to be statistically significant at *p* < 0.05 and highly significant at *p* < 0.001.

## Supplementary Materials

The following are available online at https://www.mdpi.com/1660-3397/17/7/412/s1, Figure S1: Establishment of paclitaxel-resistant ovarian cancer cells, Figure S2: Effect of GTX treatment on autophagy and the Bcl-2 family in PTX-resistant ovarian cancer cells.

## References

[1] Bialik S., Kimchi A. (2006). The death-associated protein kinases: Structure, function, and beyond. Annu. Rev. Biochem..

[2] Sanchez-Cespedes M., Esteller M., Wu L., Nawroz-Danish H., Yoo G.H., Koch W.M., Jen J., Herman J.G., Sidransky D. (2000). Gene promoter hypermethylation in tumors and serum of head and neck cancer patients. Cancer Res..

[3] Kim D.H., Nelson H.H., Wiencke J.K., Christiani D.C., Wain J.C., Mark E.J., Kelsey K.T. (2001). Promoter methylation of DAP-kinase: Association with advanced stage in non-small cell lung cancer. Oncogene.

[4] Dansranjavin T., Möbius C., Tannapfel A., Bartels M., Wittekind C., Hauss J., Witzigmann H. (2006). E-cadherin and DAP kinase in pancreatic adenocarcinoma and corresponding lymph node metastases. Oncol. Rep..

[5] Raveh T., Droguett G., Horwitz M.S., DePinho R.A., Kimchi A. (2001). DAP kinase activates a p19ARF/p53-mediated apoptotic checkpoint to suppress oncogenic transformation. Nat. Cell Biol..

[6] Yoo H.J., Byun H.J., Kim B.R., Lee K.H., Park S.Y., Rho S.B. (2012). DAPk1 inhibits NF-κB activation through TNF-α and INF-γ-induced apoptosis. Cell Signal..

[7] Wu B., Yao H., Wang S., Xu R. (2013). DAPK1 modulates a curcumin-induced G2/M arrest and apoptosis by regulating STAT3, NF-κB, and caspase-3 activation. Biochem. Biophys. Res. Commun..

[8] Zalckvar E., Berissi H., Eisenstein M., Kimchi A. (2009). Phosphorylation of Beclin 1 by DAP-kinase promotes autophagy by weakening its interactions with Bcl-2 and Bcl-XL. Autophagy.

[9] Inbal B., Bialik S., Sabanay I., Shani G., Kimchi A. (2002). DAP kinase and DRP-1 mediate membrane blebbing and the formation of autophagic vesicles during programmed cell death. J. Cell Biol..

[10] Jin Y., Gallagher P.J. (2003). Antisense depletion of death-associated protein kinase promotes apoptosis. J. Biol. Chem..

[11] Zhao J., Zhao D., Poage G.M., Mazumdar A., Zhang Y., Hill J.L., Hartman Z.C., Savage M.I., Mills G.B., Brown P.H. (2015). Death-associated protein kinase 1 promotes growth of p53-mutant cancers. J. Clin. Investig..

[12] Brady C.A., Jiang D., Mello S.S., Johnson T.M., Jarvis L.A., Kozak M.M., Kenzelmann Broz D., Basak S., Park E.J., McLaughlin M.E. (2011). Distinct p53 transcriptional programs dictate acute DNA-damage responses and tumor suppression. Cell.

[13] Itahana Y., Itahana K. (2018). Emerging Roles of p53 Family Members in Glucose Metabolism. Int. J. Mol. Sci..

[14] Martoriati A., Doumont G., Alcalay M., Bellefroid E., Pelicci P.G., Marine J.C. (2005). dapk1, encoding an activator of a p19ARF-p53-mediated apoptotic checkpoint, is a transcription target of p53. Oncogene.

[15] Marin J.J., Romero M.R., Martinez-Becerra P., Herraez E., Briz O. (2009). Overview of the molecular bases of resistance to chemotherapy in liver and gastrointestinal tumours. Curr. Mol. Med..

[16] Hientz K., Mohr A., Bhakta-Guha D., Efferth T. (2017). The role of p53 in cancer drug resistance and targeted chemotherapy. Oncotarget.

[17] Debernardis D., Siré E.G., De Feudis P., Vikhanskaya F., Valenti M., Russo P., Parodi S., D’Incalci M., Broggini M. (1997). p53 status does not affect sensitivity of human ovarian cancer cell lines to paclitaxel. Cancer Res..

[18] Petty R., Evans A., Duncan I., Kurbacher C., Cree I. (1998). Drug resistance in ovarian cancer—The role of p53. Pathol. Oncol. Res..

[19] Wei J., Zaika E., Zaika A. (2012). p53 Family: Role of Protein Isoforms in Human Cancer. J. Nucleic Acids.

[20] Dötsch V., Bernassola F., Coutandin D., Candi E., Melino G. (2010). p63 and p73, the ancestors of p53. Cold Spring Harb. Perspect. Biol..

[21] Su X., Gi Y.J., Chakravarti D., Chan I.L., Zhang A., Xia X., Tsai K.Y., Flores E.R. (2012). TAp63 is a master transcriptional regulator of lipid and glucose metabolism. Cell Metab..

[22] Flores E.R., Sengupta S., Miller J.B., Newman J.J., Bronson R., Crowley D., Yang A., McKeon F., Jacks T. (2005). Tumor predisposition in mice mutant for p63 and p73: Evidence for broader tumor suppressor functions for the p53 family. Cancer Cell.

[23] Su X., Chakravarti D., Cho M.S., Liu L., Gi Y.J., Lin Y.L., Leung M.L., El-Naggar A., Creighton C.J., Suraokar M.B. (2010). TAp63 suppresses metastasis through coordinate regulation of Dicer and miRNAs. Nature.

[24] Park G.B., Kim Y.S., Lee H.K., Yang J.W., Kim D., Hur D.Y. (2016). ASK1/JNK-mediated TAp63 activation controls the cell survival signal of baicalein-treated EBV-transformed B cells. Mol. Cell. Biochem..

[25] Park G.B., Chung Y.H., Gong J.H., Jin D.H., Kim D. (2016). GSK-3β-mediated fatty acid synthesis enhances epithelial to mesenchymal transition of TLR4-activated colorectal cancer cells through regulation of TAp63. Int. J. Oncol..

[26] Waring P., Sjaarda A., Lin Q.H. (1995). Gliotoxin inactivates alcohol dehydrogenase by either covalent modification or free radical damage mediated by redox cycling. Biochem. Pharmacol..

[27] Gardiner D.M., Waring P., Howlett B.J. (2005). The epipolythiodioxopiperazine (ETP) class of fungal toxins: Distribution, mode of action, functions and biosynthesis. Microbiology.

[28] López-Franco O., Suzuki Y., Sanjuán G., Blanco J., Hernández-Vargas P., Yo Y., Kopp J., Egido J., Gómez-Guerrero C. (2002). Nuclear factor-kappa B inhibitors as potential novel anti-inflammatory agents for the treatment of immune glomerulonephritis. Am. J. Pathol..

[29] Kim Y.S., Park S.J. (2016). Gliotoxin from the marine fungus Aspergillus fumigatus induces apoptosis in HT1080 fibrosarcoma cells by downregulating NF-κB. Fish. Aquat. Sci..

[30] Nguyen V.T., Lee J.S., Qian Z.J., Li Y.X., Kim K.N., Heo S.J., Jeon Y.J., Park W.S., Choi I.W., Je J.Y. (2013). Gliotoxin isolated from marine fungus Aspergillus sp. Induces apoptosis of human cervical cancer and chondrosarcoma cells. Mar. Drugs.

[31] Hubmann R., Hilgarth M., Schnabl S., Ponath E., Reiter M., Demirtas D., Sieghart W., Valent P., Zielinski C., Jäger U. (2013). Gliotoxin is a potent NOTCH2 transactivation inhibitor and efficiently induces apoptosis in chronic lymphocytic leukaemia (CLL) cells. Br. J. Haematol..

[32] Pan X.Q., Harday J. (2007). Electromicroscopic observations on gliotoxin-induced apoptosis of cancer cells in culture and human cancer xenografts in transplanted SCID mice. In Vivo.

[33] Manh Hung L.V., Song Y.W., Cho S.K. (2018). Effects of the Combination of Gliotoxin and Adriamycin on the Adriamycin-Resistant Non-Small-Cell Lung Cancer A549 Cell Line. Mar. Drugs.

[34] Harrison B., Kraus M., Burch L., Stevens C., Craig A., Gordon-Weeks P., Hupp T.R. (2008). DAPK-1 binding to a linear peptide motif in MAP1B stimulates autophagy and membrane blebbing. J. Biol. Chem..

[35] Katzenellenbogen R.A., Baylin S.B., Herman J.G. (1999). Hypermethylation of the DAP-kinase CpG island is a common alteration in B-cell malignancies. Blood.

[36] Esteller M., Sanchez-Cespedes M., Rosell R., Sidransky D., Baylin S.B., Herman J.G. (1999). Detection of aberrant promoter hypermethylation of tumor suppressor genes in serum DNA from non-small cell lung cancer patients. Cancer Res..

[37] Yao J.Y., Chen J.K. (2010). TAp63 plays compensatory roles in p53-deficient cancer cells under genotoxic stress. Biochem. Biophys. Res. Commun..

[38] Jang C.W., Chen C.H., Chen C.C., Chen J.Y., Su Y.H., Chen R.H. (2002). TGF-beta induces apoptosis through Smad-mediated expression of DAP-kinase. Nat. Cell Biol..

[39] Housman G., Byler S., Heerboth S., Lapinska K., Longacre M., Snyder N., Sarkar S. (2014). Drug resistance in cancer: An overview. Cancers.

[40] Sullivan G.F., Yang J.M., Vassil A., Yang J., Bash-Babula J., Hait W.N. (2000). Regulation of expression of the multidrug resistance protein MRP1 by p53 in human prostate cancer cells. J. Clin. Investig..

