# Supplementary material for: Gliotoxin Enhances Autophagic Cell Death via the DAPK1-TAp63 Signaling Pathway in Paclitaxel-Resistant Ovarian Cancer Cells
Source: Mar Drugs. 2019 Jul 12;17(7):412. doi: 10.3390/md17070412 (PMC6669733; doi:10.3390/md17070412)
Supplement: Supplementary file 1 [file marinedrugs-17-00412-s001.zip › Legends for supplemental figures(Marine drugs)_revision.docx]

**Supplementary figure legends**

**Supplemental Figure 1. Establishment of paclitaxel-resistant ovarian cancer cells.** (A) Typical fibroblast-like morphology of PTX-resistant ovarian cancer cells. Images were taken using an inverted phase-contrast microscope at ×100 magnification. (B) Cells were treated with the indicated drug concentration for 48 h. Cell viability was measured using a Cell Counting Kit-8 assay. The absorbance at 450 nm is presented. n=3. *P < 0.001 compared with DMSO. The results are representative of three independent experiments.

**Supplemental Figure 2. Effect of GTX treatment on autophagy and the Bcl-2 family in PTX-resistant ovarian cancer cells.** Cells were treated with GTX or PTX alone or in combination. Total protein from each group was subjected to Western blot analysis with the indicated antibodies. The results are representative of three independent experiments.
