# Supplementary figures and images for: Gliotoxin Enhances Autophagic Cell Death via the DAPK1-TAp63 Signaling Pathway in Paclitaxel-Resistant Ovarian Cancer Cells
Source: Mar Drugs. 2019 Jul 12;17(7):412. doi: 10.3390/md17070412 (PMC6669733; doi:10.3390/md17070412)

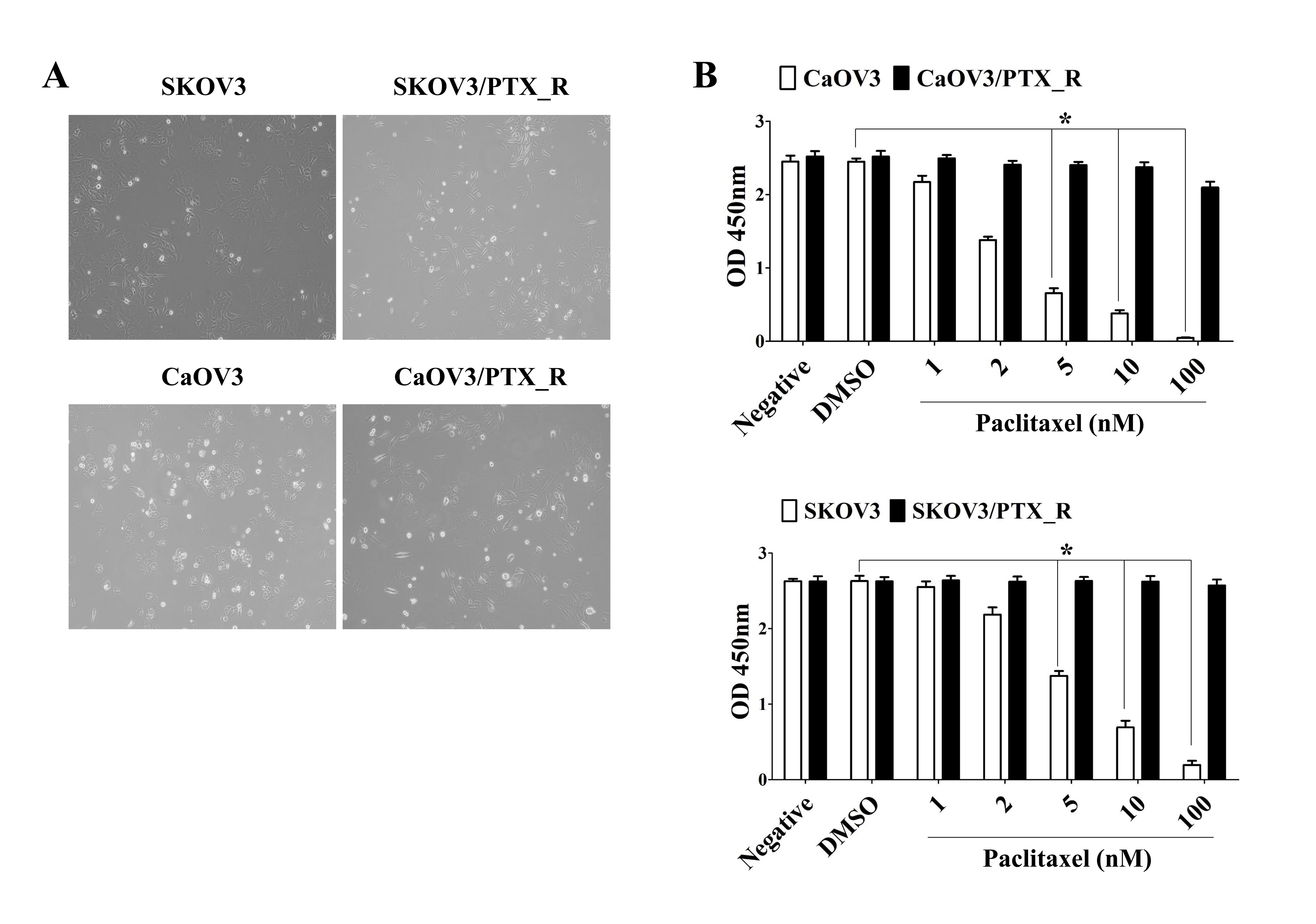

Supplement: Supplementary file 1 [file marinedrugs-17-00412-s001.zip › supple.fig.1.tif]

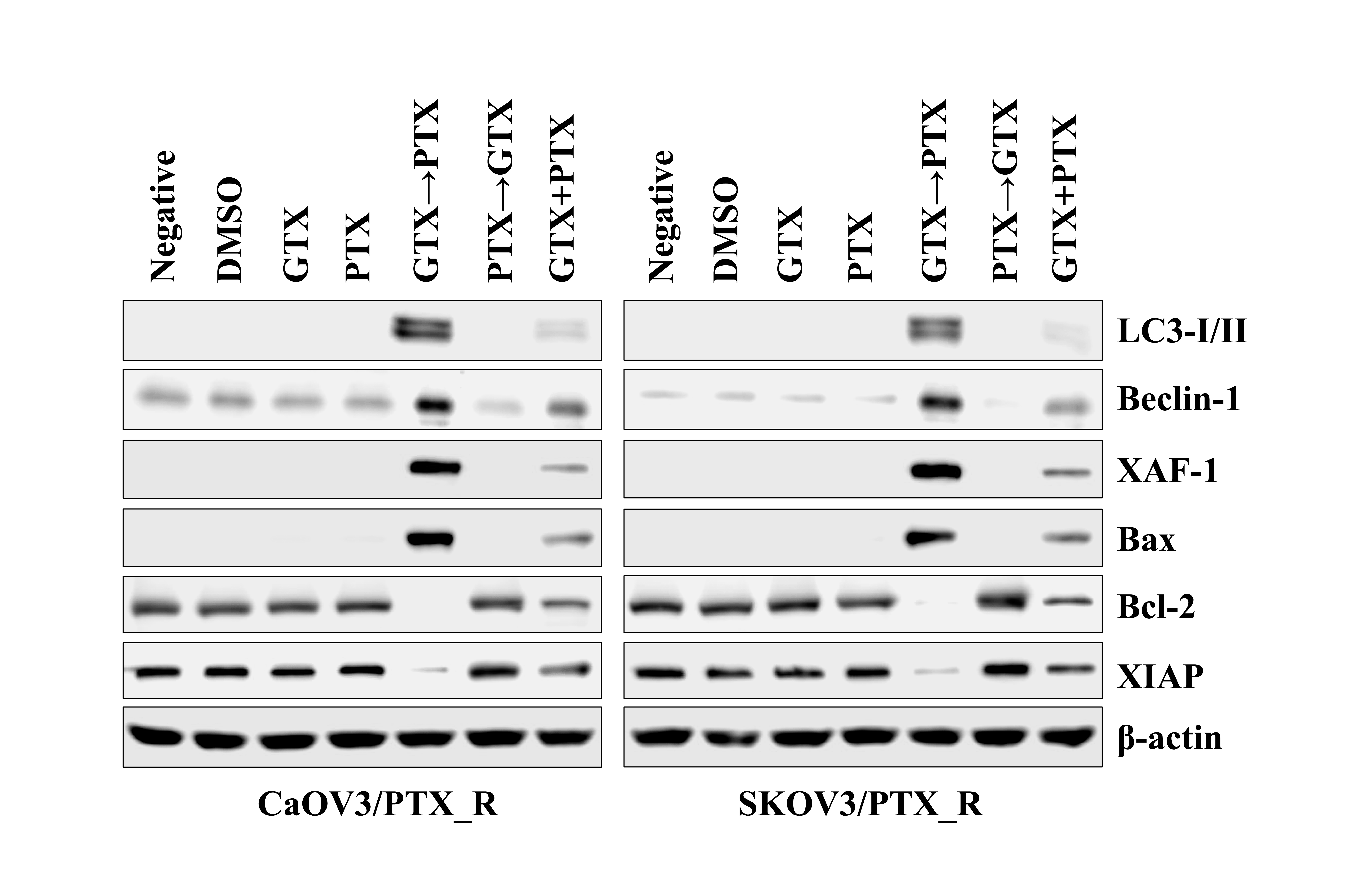

Supplement: Supplementary file 1 [file marinedrugs-17-00412-s001.zip › supple.fig.2.tif]
